# Supplementary material for: Staphylococcus spp. Causatives of Infections and Carrier of blaZ, femA, and mecA Genes Associated with Resistance
Source: Antibiotics (Basel). 2023 Mar 29;12(4):671. doi: 10.3390/antibiotics12040671 (PMC10135354; doi:10.3390/antibiotics12040671)
Supplement: Supplementary file 1 [file antibiotics-12-00671-s001.zip › antibiotics-2264779-supplementary.pdf]

Table S1. *Staphylococcus* spp. tested for chromosomal and plasmid *blaZ*, *fem*A and *mec*A genes and oxacillin and penicillin G resistance profile

Sample number	Species	Chromosomal			Plasmid			Resistance	
		blaZ	*femA*	*mecA*	*blaZ*	*femA*	*mecA*	Oxacillin	Penicillin G
1.1	*Staphylococcus haemolyticus*	-	-	-	-	-	-	Negative	Negative
1.2	*Staphylococcus intermedius*	Negative	-	-	Negative	Negative	Negative	Negative	Positive
1.3	*Staphylococcus haemolyticus*	Negative	-	Positive	-	Positive	Negative	Positive	Negative
1.4	*Staphylococcus haemolyticus*	Negative	Negative	-	Negative	Positive	Negative	Negative	Positive
1.5	*Staphylococcus aureus subsp. aureus*	Negative	Negative	Negative	Negative	Negative	Positive	Negative	Positive
1.6	*Staphylococcus aureus subsp. aureus*	-	-	-	-	-	-	Negative	Negative
1.7	*Staphylococcus haemolyticus*	Negative	Negative	Negative	Negative	Negative	Negative	Negative	Positive
1,8	*Staphylococcus haemolyticus*	Negative	Positive	Negative	Negative	Negative	Negative	Negative	Positive
1.9	*Staphylococcus carnosus subsp. carnosus*	Negative	Negative	-	Negative	Negative	Negative	Positive	Negative
1.10	*Staphylococcus haemolyticus*	Negative	Negative	Negative	Positive	Positive	Negative	Negative	Negative
1.11	Staphylococcus aureus subsp. aureus	Negative	Negative	Negative	-	Positive	Positive	Negative	Positive
1.12	*Staphylococcus carnosus subsp. carnosus*	Negative	Positive	Negative	Positive	Positive	Positive	Negative	Negative
2.1	*Staphylococcus intermedius*	Negative	Positive	Negative	Negative	Positive	Negative	Negative	Negative
2.2	*Staphylococcus haemolyticus*	Negative	Negative	Negative	Negative	Negative	Negative	Positive	Positive
2.3	*Staphylococcus haemolyticus*	Negative	-	Negative	Negative	Negative	Negative	Positive	Negative
2.4	*Staphylococcus haemolyticus*	Negative	Negative	Negative	-	Negative	Negative	Positive	Negative
2.5	*Staphylococcus haemolyticus*	Negative	-	Negative	Negative	Negative	-	Positive	Positive
2.6	*Staphylococcus haemolyticus*	Negative	-	Negative	Negative	Negative	Negative	Positive	Negative
2.7	*Staphylococcus haemolyticus*	-	Positive	Negative	Negative	Positive	Negative	Positive	Negative
2.8	*Staphylococcus haemolyticus*	Negative	-	Negative	-	Positive	Negative	Positive	Positive
2.9	*Staphylococcus aureus subsp. aureus*	Negative	Negative	Negative	Negative	Negative	Negative	Positive	Positive
3.1	*Staphylococcus haemolyticus*	-	-	-	-	-	-	Negative	Positive
3.2	*Staphylococcus haemolyticus*	-	-	-	-	-	-	Positive	Positive
3.3	*Staphylococcus agnetis*	Negative	-	Negative	Positive	-	Positive	Positive	Positive
3.4	*Staphylococcus haemolyticus*	Negative	Negative	Negative	Negative	-	-	Positive	Positive
3.5	*Staphylococcus carnosus subsp. utilis*	Negative	-	Negative	Positive	Positive	Negative	Positive	Positive
3.6	*Staphylococcus carnosus subsp. utilis*	-	-	Negative	Negative	Negative	Negative	Positive	Negative
3.7	*Staphylococcus haemolyticus*	-	Negative	Negative	-	-	Negative	Positive	Positive
3.8	*Staphylococcus auricularis*	-	-	-	-	-	-	Positive	Positive
4.1	*Staphylococcus haemolyticus*	-	-	-	-	-	-	Positive	Positive
4.2	*Staphylococcus agnetis*	Negative	-	Negative	-	-	-	Positive	Positive
4.3	*Staphylococcus haemolyticus*	-	-	-	-	-	-	Positive	Positive
4.4	*Staphylococcus auricularis*	-	-	Negative	-	-	-	Positive	Positive
4.5	*Staphylococcus auricularis*	-	-	-	-	-	-	Positive	Positive
4.6	*Staphylococcus epidermidis*	-	Positive	-	Negative	-	-	Positive	Positive
5.1	*Staphylococcus carnosus subsp. utilis*	-	-	-	-	-	-	Positive	Positive
5.2	*Staphylococcus carnosus subsp. utilis*	Negative	Positive	Negative	Negative	Positive	Positive	Positive	Positive
5.3	*Staphylococcus haemolyticus*	-	Positive	-	-	-	-	Positive	Positive
5.4	*Staphylococcus auricularis*	-	-	Negative	-	-	-	Negative	Negative

5.7	*Staphylococcus agnetis*	-	Negative	-	-	-	-	Positive	Positive
5.8	*Staphylococcus auricularis*	Negative	Positive	Negative	Negative	-	Negative	Positive	Positive
6.1	*Staphylococcus pasteuri*	Negative	Positive	Negative	Negative	Negative	Negative	Negative	Positive
6.2	*Staphylococcus intermedius*	Negative	Positive	Negative	Negative	Positive	Negative	Positive	Positive
6.3	*Staphylococcus intermedius*	Negative	Positive	Negative	Negative	Negative	Negative	Positive	Positive
6.4	*Staphylococcus intermedius*	Negative	Positive	-	Negative	Negative	Positive	Positive	Negative
6.5	*Staphylococcus intermedius*	Negative	Positive	Negative	Negative	Negative	Negative	Negative	Negative
8.1	*Staphylococcus auricularis*	Negative	Negative	Negative	Positive	Positive	Negative	Positive	Positive
8.2	*Staphylococcus intermedius*	Negative	Positive	Negative	Positive	Positive	Negative	Positive	Positive

Legend: - represent untested samples for a given gen

Table S2. Logistic regression to verify the association between the presence of the gene and resistance to penicillin G and oxacillin.

	Gene	Penicillin G - Resistant			Oxacillin - Resistant		
		OR	IC-95%	p-valor	OR	IC-95%	p-valor
All species	blaZ-c	*	*	*	*	*	*
	femA-c	0.78	(0.09; 6.79)	0.51	1.53	(0.25; 9.55)	0.55
	mecA-c	438676.90	(0.0; 1.17)	0.96	117878.60	(0.0; 3.13)	0.74
	blaZ-p	2.00	(0.07; 54.72)	0.52	0.97	(0.07; 12.47)	0.94
	femA-p	265873.42	(0.0; 2.30)	0.45	0.97	(0.12; 7.11)	0.61
	mecA-p	6.47	(0.07; 641.61)	0.73	0.48	(0.06; 3.71)	0.71
S. aureus subsp. *aureus*	femA-p	1.00	(0.0; 3.15)	0.99	0.00	(0.0; 9.28)	1.00
	mecA-p	1.00	(0.0; 3.51)	0.99	0.00	(0.0; 2.34)	0.99
S. auricularis	femA-c	1.00	(0.0; 1.28)	1.00	1.00	(0.0; 1.28)	1.00
	blaZ-p	1.00	(0.0; 1.28)	0.87	1.00	(0.0; 1.28)	1.00
S. carnosus subsp. *utilis*	femA-c	*	*	*	0.00	(0.0; 4.32)	0.90
	blaZ-p	286663.11	(0.0; 7.63)	1.00	0.00	(0.0; 4.32)	0.90
	femA-p	1.50	(0.0; 5.30)	1.00	0.00	(0.0; 4.32)	0.90
	mecA-p	*	*	*	0.00	(0.0; 4.32)	0.90
S. haemolyticus	femA-c	0.96	(0.0; 2.61)	0.96	2.88	(0.09; 88.34)	0.61
	mecA-c	0.00	(0.0; 5.24)	0.99	59848.83	(0.0; 1.60)	1.00
	blaZ-p	0.00	(0.0; 1.84)	0.97	*	*	*
	femA-p	5.00	(0.0; 4.68)	0.94	0.82	(0.03; 19.75)	0.99
S. intermedius	blaZ-p	105456.47	(0.0; 4.86)	0.95	286663.12	(0.0; 7.63)	0.97
	femA-p	1.00	(0.02; 50.40)	1.00	1.00	(0.2; 50.40)	1.00
	mecA-p	*	*	*	286663.12	(0.0; 7.63)	0.97

*did not have enough data to calculate such estimates.

-c (chromosomal DNA)

-p (plasmid DNA)

Table S3. X^2 test for association between the presence of genes and resistance to penicillin G and oxacillin.

	Gene	X^2	p-valor
Oxacillin	femA-c	1854.00	0.396
	blaZ-p	0.19	0.909
	femA-p	0.70	0.705
	mecA-p	0.89	0.642
Penicillin G	femA-c	0.58	0.75
	blaZ-p	0.64	0.725
	femA-p	3.59	0.166
	mecA-p	2.37	0.306

-c (chromosomal DNA)

-p (plasmid DNA
